# Timing of Wound Dressing Removal After Emergency Cesarean Section: A Randomized Controlled Trial

**DOI:** 10.7759/cureus.104106

**Published:** 2026-02-23

**Authors:** Zahar A Zakaria, Mazrin N Mohd Ali, Fadzlin Mohd Adzlan, Anis S Musa

**Affiliations:** 1 Obstetrics and Gynecology, Hospital Kemaman, Chukai, MYS

**Keywords:** cesarean delivery, emergency cesarean, surgical site complication, wound complication, wound dressing

## Abstract

Introduction

Previously, cesarean wound dressing was usually removed 48 hours after surgery, but recent data have shown that early removal is not detrimental. However, this practice is derived mainly from trials involving scheduled cesarean delivery. This study was designed to investigate the effect of wound dressing removal after emergency cesarean section during labor.

Materials and methods

The study was a randomized controlled trial recruiting women with a singleton, term pregnancy in the active phase of labor. The main objective was to determine the incidence of wound complications when the wound dressing was removed at 24 and 48 hours after surgery. Other outcomes of interest were the maximum pain score and the timing of showering after cesarean delivery.

Results

A total of 294 women were initially recruited, and data for 291 were available for final analysis. There was no significant difference in the general, labor, and surgical characteristics. The incidence of wound complications was 4.1% and 7.5% (OR 0.53, 95% CI 0.19-1.5; p = 0.217) in women who had dressing removal at 24 and 48 hours after surgery, respectively. There was also no difference in maximum pain score after surgery or timing of showering related to wound dressing removal.

Conclusions

Early wound dressing removal, at 24 hours after surgery, in women who had emergency cesarean delivery during labor, is not associated with an increased incidence of wound complications. This result could support the practice of early discharge after cesarean delivery.

## Introduction

The cesarean section rate worldwide is showing an increasing trend, from below 7% in 1990 to 19% in 2014 [1]. In 2022, cesarean delivery accounted for 32.1% of all births in the United States, numbering almost 1.2 million [2]. In Malaysia, more than 30,000 cesarean sections were reported in 2020, comprising 29.6% of all deliveries [3]. An increasing number of cesarean deliveries will invariably lead to a higher number of complications, and one of the most common, as with other types of surgeries, is wound complications, which include surgical wound infection. Surgical site infection (SSI) significantly increases hospital stay and may even have an emotional impact on the patient, more so for a postpartum mother who is already stressed by the delivery process and the care of the newborn [4]. It is estimated that the cost of treating SSI after cesarean delivery may reach USD 3,700 per person [5].

In a clean surgical wound, epithelialization typically occurs in the first 48 hours following surgery, creating a barrier to bacteria and other foreign bodies. However, the superficial epithelium is very thin, easily traumatized, and has low tensile strength, hence the need for a cover in the form of a surgical dressing [6]. The purpose of surgical dressing is also to absorb exudate, reduce exposure of the wound to pathogens, and protect the wound until the skin restores its integrity [7]. Dressing also promotes wound healing by creating and maintaining a warm and moist environment [8].

Surgical wound dressings are usually left in place for 24-48 hours, allowing the development of a moist environment, which has been shown to cause quicker healing compared to a dry wound. However, there are conflicting findings, where excess exudate may cause softening or deterioration of the wound and may accelerate microorganism growth. This raises questions about the ideal timing of wound dressing removal.

Randomized controlled trials have shown that the proportion of wound complications or infection is similar when the dressing is removed earlier or later [9-11]. These studies recruited mainly women who underwent planned cesarean delivery, leaving a void in data related to unscheduled surgeries. We conducted a randomized controlled trial to investigate the effect of early versus late wound dressing removal (after 24 hours versus after 48 hours of surgery) in women who underwent emergency lower-segment cesarean delivery during labor.

## Materials and methods

This randomized controlled trial, conducted from August 1, 2022, to December 31, 2024, involved women aged 18 to 45 years with a singleton pregnancy at term who underwent emergency cesarean delivery in the active phase of labor. Women were excluded if they had an upper segment or classical cesarean section, had massive postpartum hemorrhage of more than 1.5 liters, or had interrupted sutures for skin closure. Other exclusion criteria were predelivery chorioamnionitis, acquired or congenital coagulation disorders, and/or any medical or obstetric condition requiring prolonged hospital stay after delivery, such as severe preeclampsia, uncontrolled diabetes mellitus, cardiac disease, or systemic infection.

The primary objective was to determine the incidence of cesarean wound complications, which include hematoma, seroma, infection, and wound dehiscence. Seroma and hematoma are defined as collections of serous fluid and blood, respectively, while wound dehiscence is the separation of the margins of the surgical incision, with or without exposure or protrusion of underlying tissue, organs, or implants [12,13]. Wound infection includes superficial or deep incisional and organ or surgical space infections that occur up to 30 days after the cesarean section [4]. The secondary objectives were the maximum pain score after surgery, assessed daily using a Visual Analogue Scale of 10 before discharge, and the timing of showering after cesarean delivery. The participants’ general characteristics and data related to labor and cesarean section were also analyzed and compared between the study groups. This trial methodology was based on the Consolidated Standards of Reporting Trials statement [14].

All cesarean sections in our center, a major specialist hospital with an annual delivery rate of about 4,000, receive a dose of intravenous amoxicillin-clavulanic acid as surgical prophylaxis within 30 minutes before surgery. The abdominal skin is cleaned with chlorhexidine 1:200 (0.5%) in 70% alcohol, while povidone-iodine 10% in diluted form is used to swab the upper vagina. All cesarean sections are performed according to the Joel-Cohen or modified Misgav Ladac method, and the lower uterine segment is closed in two layers using No. 1 polyglycolic acid suture, while the peritoneal layers are left unopposed. The rectus fascia is closed using a continuous non-locking method with No. 1 polyglycolic acid suture, and the subcutaneous tissue is approximated using 2/0 rapidly absorbing polyglycolic acid suture if the thickness is more than 2 cm. In this study, 3/0 rapidly absorbing polyglycolic acid suture was used for subcuticular skin closure. All wounds were then cleaned with normal saline prior to skin approximation and covered with 3M™ Soft Cloth Dressing with Pad (Wellkang Ltd, Dover, UK).

During the trial, within 12 hours post-surgery or after regaining full consciousness, eligible women were counseled by one of the researchers. Informed consent was obtained from potential study candidates, and those who consented had the wound exposed after 24 or 48 hours of surgery, according to the randomized intervention. All participants received standard post-cesarean oral analgesia, either a combination of paracetamol and diclofenac sodium or tramadol.

Postoperative management involved early ambulation, early removal of the indwelling bladder catheter, and the administration of subcutaneous venous thromboprophylaxis for a total of 10 days after surgery (based on the latest local clinical practice guideline) [15]. Showering was permitted once the patients started to ambulate, and the timing was left to their own preference. All patients were discharged 48 hours after surgery if no further monitoring or inpatient management was required.

Postpartum women received home visits by primary health care personnel on days 4, 6, 8, 10, 15, and 20 after delivery, and any wound complication was reported to the research team. These women were also contacted by telephone after the 30th day of surgery by one of the researchers to inquire about any wound complications that might not have been reported earlier.

A previous trial studying scheduled cesarean delivery found that the average incidence of wound complications was 13.75% [9]. As our study included emergency surgeries, we estimated that the wound complication rate would be similar or higher. To demonstrate a 100% increase in wound complications from the estimated incidence of 13% with 80% power and a 95% confidence interval, a total of 294 participants were required. Randomization was performed in blocks of ten with a 1:1 ratio, with numbers generated using a computerized random number generator (www.random.org). Blinding was ensured using sealed opaque envelopes containing the randomization number and instructions on wound dressing removal. Mean and standard deviation or median and quartiles were calculated for quantitative variables and analyzed using the Student’s t-test or Mann-Whitney U test, while qualitative data were reported as percentages and analyzed using the chi-square test or Fisher’s exact test when necessary. Data handling and analysis were performed using IBM SPSS Statistics for Windows, Version 22.0 (Released 2013; IBM Corp., Armonk, NY, USA), with a p-value of less than 0.05 considered to indicate statistical significance.

This study was approved by the Medical Research & Ethics Committee, Ministry of Health Malaysia, and registered at ClinicalTrials.gov (NCT05458518). It was conducted in compliance with the Declaration of Helsinki on human experimentation and Malaysian Good Clinical Practice Guidelines [16].

## Results

**Figure 1:**
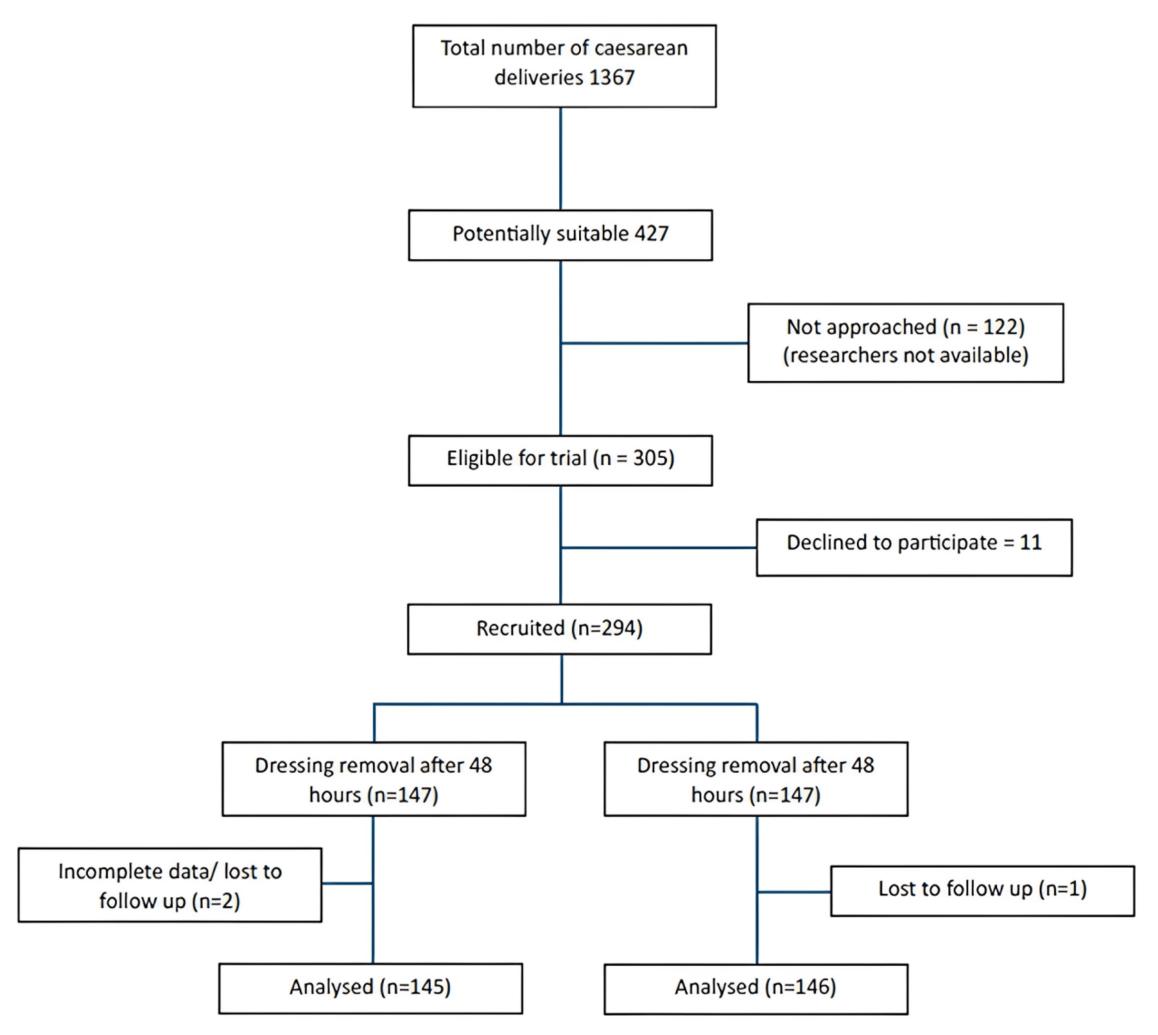
Recruitment flowchart of study participants

During the 48-month recruitment period, there were 7,884 deliveries, with 1,367 cesarean sections, in our hospital. The final number required for this trial (294) was achieved after excluding 122 potential participants due to the researchers’ nonavailability. Only three patients were excluded from the final analysis due to incomplete data or loss to follow-up. The recruitment flowchart of study participants is shown in Figure 1.

There was no significant difference in the general and labor characteristics of the participants in both groups (Table 1). The perioperative findings were also similar among these women (Table 2). Suspected fetal compromise was the most common indication for emergency cesarean delivery (75.1%), followed by poor labor progression, secondary arrest, and prolonged second stage of labor (17.5%). In total, 96.2% of the patients had amniorrhexis, and the majority underwent amniotomy for labor induction or augmentation. There was no difference in active labor duration before cesarean delivery between the study groups, and the mean duration for the study participants was 325.5 (± 235.5) minutes. All women completed at least 10 days of subcutaneous venous thromboembolism prophylaxis, with almost 95% using unfractionated heparin (UFH) (Table 2).

**Table 1:** Participant and labor characteristics A p-value of < 0.05 indicates statistical significance. ROM, rupture of membrane

Variable	24 hours (n = 145)	48 hours (n = 146)	p-Value
Age (year, mean ± SD)	28.8 ± 4.9	29.4 ± 4.9	t = -0.924, p = 0.356
Gravida (mean ± SD)	2.0 ± 1.5	2.0 ± 1.3	t = -0.039, p = 0.969
Gestation (week, mean ± SD)	38.8 ± 1.3	38.8 ± 1.6	t = -0.167, p = 0.867
BMI before delivery (kg/m², mean ± SD)	31.1 ± 6.1	31.0 ± 6.8	t = -0.198, p = 0.843
Obesity (n, %)	79 (54.5)	80 (54.8)	χ² = 0.003, p = 0.957
Diabetes (n, %)	50 (34.5)	47 (32.2)	χ² = 0.172, p = 0.678
Hypertension (n, %)	16 (11.0)	10 (6.8)	χ² = 1.566, p = 0.211
Labor duration (min, mean ± SD)	344.2 ± 192.5	306.8 ± 271.2	t = 1.354, p = 0.177
ROM (n, %)	138 (95.2)	142 (97.3)	χ² = 0.872, p = 0.350
Amniotomy (n, %)	97 (66.9)	90 (61.6)	χ² = 0.874, p = 0.350
ROM duration (min, mean ± SD)	530.3 ± 563.3	558.5 ± 812.5	t = -0.344, p = 0.731

**Table 2:** Operative characteristics A p-value of < 0.05 indicates statistical significance. CS, cesarean section; NSAID, non-steroidal anti-inflammatory drug; UFH, unfractionated heparin

Variable	24 hours (n = 145)	48 hours (n = 146)	p-Value
Primary CS (n, %)	123 (84.8)	114 (78.1)	χ² = 2.190, p = 0.139
Fetal distress (n, %)	102 (70.3)	106 (72.6)	χ² = 0.182, p = 0.670
Failure to progress labor (n, %)	23 (15.9)	28 (19.2)	χ² = 0.553, p = 0.457
Length of operation (min, mean ± SD)	43.7 ± 10.3	45.4 ± 14.1	t = -1.112, p = 0.267
Estimated blood loss (ml, mean ± SD)	543.8 ± 273.6	579.2 ± 345.1	t = -0.971, p = 0.333
Spinal anesthesia (n, %)	139 (95.9)	141 (96.6)	χ² = 0.102, p = 0.750
Intraoperative complication (n, %)	17 (11.7)	22 (15.1)	χ² = 0.701, p = 0.402
Blood transfusion (n, %)	2 (1.4)	4 (2.7)	χ² = 0.667, p = 0.414
NSAIDs (n, %)	138 (95.2)	130 (89.0)	χ² = 3.757, p = 0.053
UFH (n, %)	137 (94.5)	139 (94.8)	χ² = 0.078, p = 0.780

The incidence of wound complications among all women was 5.8% and was lower in the early dressing removal group (4.1% vs. 7.5%; OR 0.53, 95% CI 0.19-1.5; p = 0.217), but the difference was not statistically significant. One case of deep wound infection was reported in the 48-hour dressing removal group. Approximately 70% of wound complications were detected or reported by the 10th postpartum day, and the median time of detection was 10 days after surgery (range two to 22 days).

The maximum recorded pain score after surgery was low and similar between the groups (2.9 vs. 3.1; OR 0.53, 95% CI 0.19-1.5; p = 0.217). About half of the women in both study groups took their first shower after dressing removal, and the difference was not statistically significant (Table 3).

**Table 3:** Primary and secondary outcomes A p-value of < 0.05 indicates statistical significance. SSI, surgical site infection

Variable	24 hours (n = 145)	48 hours (n = 146)	p-Value
Wound complication (n, %)	6 (4.1)	11 (7.5)	χ² = 1.526, p = 0.217
Seroma (n, %)	1 (16.7)	4 (36.4)	-
Hematoma (n, %)	2 (33.3)	4 (36.4)	-
Superficial SSI (n, %)	3 (50.0)	2 (18.2)	-
Deep SSI (n, %)	0	1 (9.1)	-
Time detected post-surgery (days, mean ± SD)	8.7 ± 5.9	11.9 ± 5.3	t = -1.618, p = 0.264
Maximum pain score (mean ± SD)	2.9 ± 1.4	3.1 ± 1.3	t = -1.103, p = 0.271
Shower after wound dressing removal (n, %)	82 (56.6)	72 (49.3)	χ² = 1.529, p = 0.216
Interval surgery-shower (hours, median (IQR))	27.2 (15.1-34.1)	39.1 (19.4-48.5)	Mann-Whitney U = 6947.00, p < 0.001

## Discussion

The incidence of SSI after cesarean section ranges from 1.5% to 9.5%, while wound complications, which include hematoma and seroma, are higher at 4.4% to 12.5% [6,13,16-19]. These wide discrepancies are likely due to variations in population characteristics, perioperative interventions, and infection classification.

The timing of wound dressing removal has been a subject of interest, as physiological changes in wound healing suggest that the wound requires protection during the first few days after surgery. To date, there is no consensus among professional bodies on the optimal timing for surgical dressing removal, including after cesarean delivery. The Centers for Disease Control and Prevention recommended in 1999 that dressings be kept in place for 24-48 hours, but the latest guideline no longer specifies a recommendation [4,20]. The National Institute for Health and Care Excellence (UK), on the other hand, recommends exposing the cesarean wound six to 24 hours after surgery [21]. Local national guidelines suggest that the wound should remain covered for 24-72 hours postoperatively [22,23].

Previous randomized controlled trials have shown no difference in wound complication or infection rates when dressings are removed early or later (or even when left uncovered), except for a study in Turkey, which reported that removing the dressing after 48 hours was associated with a significantly lower wound score compared with earlier removal [9-11]. However, these studies focused primarily on scheduled cesarean deliveries, and most excluded emergency surgeries, a factor previously identified as a risk for SSI [24,25].

Our data, gathered from women who underwent emergency cesarean delivery, demonstrated no difference in wound complications whether the dressing was removed early or later. Notably, the majority of participants had ruptured amniotic membranes before delivery, another known risk factor for SSI [24]. All participants also received prophylactic UFH or low molecular weight heparin for at least 10 days after surgery, which may further increase the risk of wound complications.

The perioperative procedures in our hospital are broadly similar to those reported in other randomized controlled trials, yet the incidence of wound complications was low. Another study in Malaysia also reported a relatively low incidence of wound infection, which may be explained by population characteristics [10].

The timing of showering is not known to influence wound healing or complications, although one author suggested it should be delayed until 48 hours after surgery [25,26]. In our hospital, there is no policy on the timing of showering after cesarean delivery. In this study, 52.9% of participants showered after wound dressing removal. There was no difference in wound complications based on whether showering occurred before or after dressing removal. Overall, the mean interval between surgery and first shower was 30.9 hours (range 6.0-59.1 hours), reflecting local practices of frequent body washing due to the hot and humid climate. The incidence of wound complications was identical between women who showered before or after dressing removal (8/137 (5.8%) vs. 9/154 (5.8%)).

These findings, showing no increased incidence of wound complications with early dressing removal, support the practice of early discharge 24-28 hours after cesarean delivery. This practice could potentially improve the psychosocial well-being of patients and reduce costs associated with inpatient care [27]. While early discharge may not be critical in all facilities, in a busy hospital with high bed occupancy, such as ours, releasing patients earlier could help alleviate ward overcrowding, potentially improving patient satisfaction and providing financial benefits to all stakeholders.

As with any clinical trial, this study has limitations. It was conducted at a single center, and all participants were of Malay ethnicity, which may limit generalizability to other institutions and populations. Additionally, the observed rate of wound complications was lower than initially anticipated, so the results should be interpreted with caution.

## Conclusions

Early wound dressing removal, at 24 hours after surgery, in women who underwent emergency cesarean delivery during labor, is not associated with an increased incidence of wound complications. These findings could support the practice of early discharge after cesarean delivery, with potential benefits for patients, their families, and the healthcare system.
